# ﻿A new stream treefrog of the genus *Hyloscirtus* (Amphibia, Hylidae) from the Río Negro-Sopladora National Park, Ecuador

**DOI:** 10.3897/zookeys.1141.90290

**Published:** 2023-01-19

**Authors:** Juan C. Sánchez-Nivicela, José M. Falcón-Reibán, Diego F. Cisneros-Heredia

**Affiliations:** 1 Universidad San Francisco de Quito USFQ, Colegio de Ciencias Biológicas y Ambientales COCIBA, Instituto de Biodiversidad Tropical IBIOTROP, Laboratorio de Zoología Terrestre, Museo de Zoología, Quito 170901, Ecuador Universidad San Francisco de Quito Quito Ecuador; 2 Universidad Nacional de Colombia, Facultad de Ciencias, Grupo de Investigación Evolución y Ecología de Fauna Neotropical, Bogotá D.C., Colombia Universidad Nacional de Colombia Bogotá Colombia; 3 Instituto Nacional de Biodiversidad, División de Herpetología, Quito, Ecuador Instituto Nacional de Biodiversidad Quito Ecuador; 4 Universidad Complutense de Madrid, Facultad de Ciencias Biológicas, Madrid, España Universidad Complutense de Madrid Madrid Spain

**Keywords:** Hylid frogs, *Hyloscirtuslarinopygion* species group, Morona-Santiago, mountain forest, new species, taxonomy

## Abstract

Recent surveys in the Río Negro-Sopladora National Park revealed a striking new species of *Hyloscirtus*. The new species is easily diagnosed from all other congeners by its large body size (64.9 mm SVL in adult female); broad dermal fringes in fingers and toes; prepollex not projected into a prepollical spine and hidden under thenar tubercle; dorsum greyish-green, with paler-hued reticulum, yellow spots and black speckles; throat, venter, flanks and hidden surfaces of limbs golden-yellow with large black blotches and spots; fingers, toes and webbing yellow with black bars and spots; iris pale pink with black periphery. It is currently known only from its type locality, in the high montane forest on the southern slopes of the Cordillera Oriental of the Andes, southeastern Ecuador. The new species might be related to the *H.larinopygion* species group based on its morphology.

“*In a stream in the forest there lived a*Hyloscirtus. *Not a nasty, dirty stream, with spoor of contamination and a muddy smell, nor yet a dry, bare, sandy stream with nothing in it to perch on or to eat: it was a*Hyloscirtus-*stream, and that means environmental quality.*”

(adapted from the opening of “The Hobbit” by J. R. R. Tolkien)

## ﻿Introduction

The genus *Hyloscirtus* Peters, 1882 currently includes 39 species of stream-breeding treefrogs, representing a distinct group of riverine amphibians distributed from Costa Rica to Bolivia (Faivovich et al. 2005; Frost 2022). Broad dermal fringes on fingers and toes are synapomorphic for *Hyloscirtus*, and molecular evidence supports their monophyly (Faivovich et al. 2005; Almendáriz et al. 2014; Rivera-Correa et al. 2016; Ron et al. 2018; Reyes-Puig et al. 2022). Three monophyletic species groups have been recognised inside *Hyloscirtus*: *Hyloscirtusarmatus* group, *Hyloscirtusbogotensis* group, and *Hyloscirtuslarinopygion* group (Faivovich et al. 2005).

The *Hyloscirtuslarinopygion* species group has been diagnosed by having a large body size (SVL>60 mm) and dark brown or grey dorsum with pale marks (Duellman et al. 2016; Ron et al. 2018). Two strongly supported clades are identified within this species group, showing latitudinal replacement among each other and sympatry in central and southern Ecuador (Almendáriz et al. 2014; Rivera-Correa et al. 2016; Rojas-Runjaic et al. 2018; Ron et al. 2018; Reyes-Puig et al. 2022). Fourteen species distributed across the Andes of southern Colombia and southern Ecuador are part of the northern clade of the *H.larinopygion* species group. The southern clade currently includes four species inhabiting the Andes of southern Ecuador and northern Peru (Ron et al. 2018; Reyes-Puig et al. 2022).

Seven species of the northern clade and three species of the southern clade (marked with asterisks) of the *H.larinopygion* group occur in restricted ranges across mountain forests on the eastern Andean slopes of Colombia and Ecuador, above 2000 m elevation: *Hyloscirtuscondor Almendáriz, Brito-M., Batallas-R. & Ron, 2014; *H.hillisi Ron, Caminer, Varela-Jaramillo & Almeida-Reinoso, 2018; *H.lindae* (Duellman & Altig, 1978); *H.pacha* (Duellman & Hillis, 1990); *H.pantostictus* (Duellman & Berger, 1982); *H.psarolaimus* (Duellman & Hillis, 1990); *H.sethmacfarlanei* Reyes-Puig, D. Recalde, F. Recalde, Koch, Guayasamin, Cisneros-Heredia, Jost & Yánez-Muñoz, 2022; *H.staufferorum* (Duellman & Coloma, 1993); *H.tapichalaca (Kizirian, Coloma & Paredes-Recalde, 2003); and *H.tigrinus* Mueses-Cisneros & Anganoy-Criollo, 2008.

Recent expeditions to the Río Negro-Sopladora National Park, on the eastern slopes of the Andes of southeastern Ecuador, resulted in the discovery of a magnificent new species of *Hyloscirtus*. Herein, we describe this new species based on its distinctive morphology and colouration.

## ﻿Materials and methods

Fieldwork was carried out between February and March 2020 at the Río Negro-Sopladora National Park, on the border between the provinces of Morona-Santiago and Azuay, southeastern Andes of Ecuador. Surveyed ecosystems included paramo grasslands and montane and foothill evergreen forests, between 1000 and 3400 m elevation on the River Paute basin. We used the complete species inventory field methodology (Angulo et al. 2006), with nocturnal surveys carried out between 19:00 and 23:00. Field coordinates were obtained using a Garmin Handheld Navigator GPS and are referenced to datum WGS84.

The specimen was euthanised with a 5% lidocaine solution, fixed in 10% formalin, and preserved in 70% ethanol, following recommendations by McDiarmid (1994) and Simmons and Muñoz Saba (2005). All procedures in this study comply with the guidelines for managing live amphibians and reptiles in field investigations (Beaupre et al. 2004). The study was carried out under scientific research authorisation N° 019-2018-IC-FAU-DNB/MAE and framework contract for access to genetic resources N° MAE-DNB-CM-2018-0106.

We reviewed diagnostic characters used for the taxonomy of the *Hyloscirtuslarinopygion* species group based on data obtained from the direct study of specimens, photographs of preserved and live frogs with verified identification from Anfibios del Ecuador BioWeb database (Ron et al. 2019), CalPhotos (Berkeley Natural History Museums 2012) and MCZbase (Museum of Comparative Zoology 2022); and from the literature, including original descriptions (Duellman 1973; Duellman and Altig 1978; Duellman and Berger 1982; Ruiz-Carranza and Lynch 1982; Duellman and Hillis 1990; Ardila-Robayo et al. 1993; Duellman and Coloma 1993; Faivovich et al. 2005; Faivovich and De la Riva 2006; Mueses-Cisneros and Anganoy-Criollo 2008; Coloma et al. 2012; Rivera-Correa and Faivovich 2013; Almendáriz et al. 2014; Duellman et al. 2016; Rivera-Correa et al. 2016; Rojas-Runjaic et al. 2018; Ron et al. 2018; Yánez-Muñoz et al. 2021; Reyes-Puig et al. 2022). The following specimens were examined for comparisons and are deposited in the following scientific collections: Museo de Zoología, Universidad San Francisco de Quito, Quito (**ZSFQ**); División de Herpetología, Instituto Nacional de Biodiversidad, Quito (**DHMECN**); Museo de Zoología, Pontificia Universidad Católica del Ecuador, Quito (**QCAZ**): *Hyloscirtuscondor*: Cerro Plateado, Zamora-Chinchipe, Ecuador (QCAZ-65235–7). *Hyloscirtuscriptico*: Cuellaje, Imbabura, Ecuador (QCAZ 42149). *Hyloscirtushillisi*: El Quimi, Morona-Santiago, Ecuador (QCAZ68655–56). *Hyloscirtuslarinopygion*: Moran, Carchi, Ecuador (DHMECN 3799). *Hyloscirtuslindae*: Parque Nacional Sumaco, Napo, Ecuador (ZSFQ 812); Sendero Oyacachi–El Chaco, Napo, Ecuador (2633–35); Guango Lodge, Napo, Ecuador (DHMECN 12483). *Hyloscirtuspacha*: Vía Gualaceo-Limón, Morona-Santiago (QCAZ 10489). *Hyloscirtuspantostictus*: Santa Barbara, Sucumbíos, Ecuador (ZSFQ 2147, 2188), La Bonita, Sucumbíos, Ecuador (ZSFQ 2187). *Hyloscirtuspsarolaimus*: Parque Nacional Sumaco, Napo, Ecuador (ZSFQ 844). *Hyloscirtusptychodactylus*: Pilaló, Cotopaxi, Ecuador (KU 209781). *Hyloscirtusstaufferorum*: Parque Nacional Sumaco, Napo, Ecuador (ZSFQ 854, 55). *Hyloscirtustapichalaca*: Tapichalaca, Zamora-Chinchipe, Ecuador (QCAZ 17776).

Format, definitions, and terminology used for the species description follow standards proposed by Duellman (1970) and Duellman and Hillis (1990). Webbing formulae follow the notation system proposed by Savage and Heyer (1967) and Myers and Duellman (1982). We use the definitions and terminology for the colouration patterns of body and limbs proposed by Savage (2002) and for eye colouration descriptions by Glaw and Vences (1997). Sex and maturity were determined by inspection of gonads through a dorsolateral incision. The following measurements were taken with digital callipers (0.01 mm accuracy, rounded to the nearest 0.1 mm) under a stereomicroscope by a single person: Snout-vent length (**SVL**), head length (**HL**), head width (**HW**), internarial distance (**IND**), interorbital distance (**IOD**), eye width (**EW**), eye-nostril distance (**EN**), eye diameter (**ED**), tympanum diameter (**TD**), tibial length (**TL**), foot length (**FL**), disc of Finger III width (**Fin3DW**). Colouration patterns in life and other relevant characteristics were obtained from field notes and photographs taken in the field.

## ﻿Results

**Figure 1.**
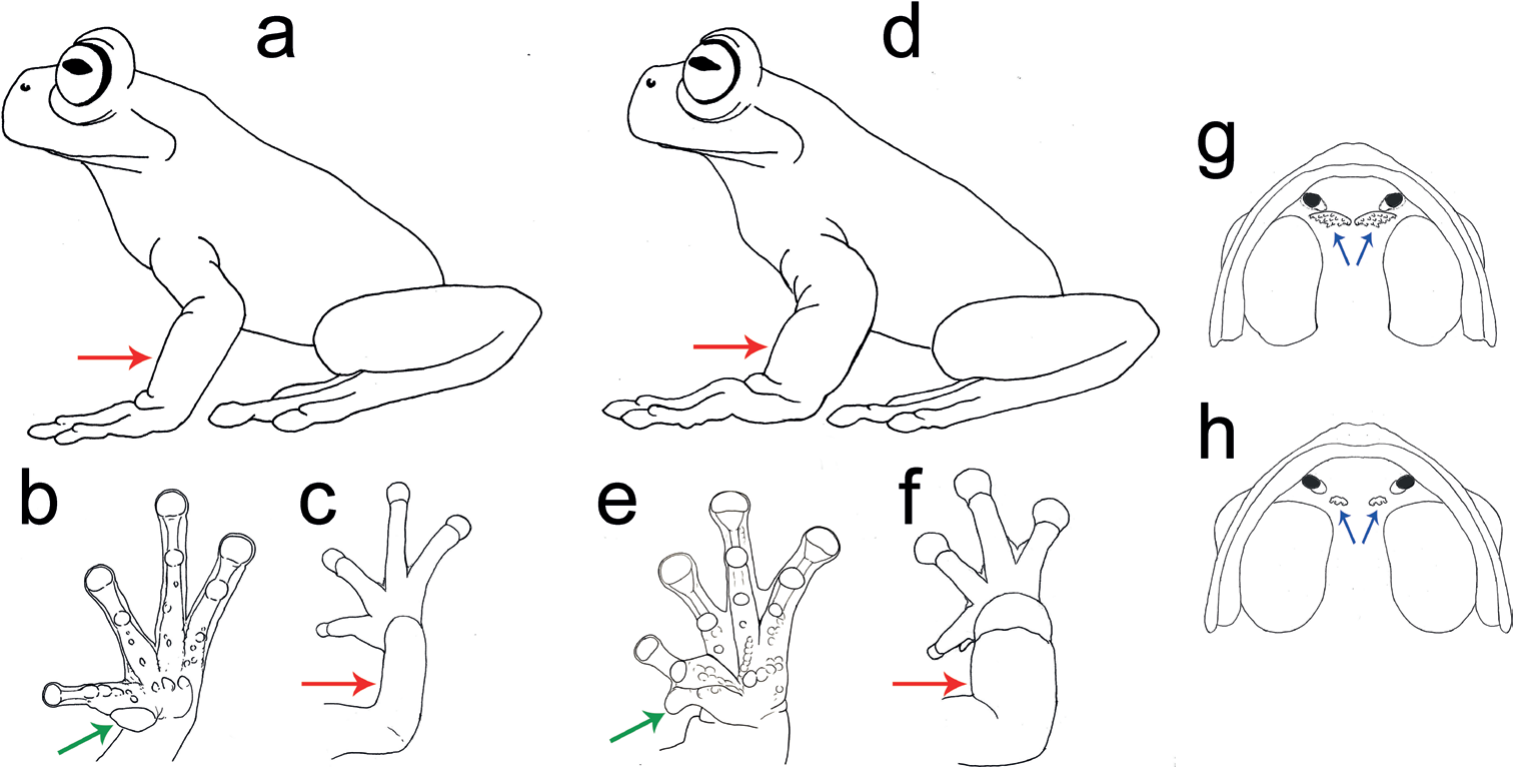
General morphology of species of the northern (**a–c, g**) and southern (**d–f, h**) clades of the *Hyloscirtuslarinopygion* species group. Red arrows in **a** and **c** show non-hypertrophied forearms, while **d** and **f** show hypertrophied forearms. Green arrow in **b** shows prepollex hidden under thenar tubercle, while **e** shows prepollex protruding in a prepollical spine. Blue arrows in **g** show dentigerous processes of vomer slightly separated with numerous vomerine teeth, while **h** show dentigerous processes of vomer notoriously separated with few vomerine teeth. Illustrations by José M. Falcón-Reibán and Juan C. Sánchez-Nivicela.

The specimen collected at Río Negro-Sopladora National Park has broad dermal fringes in fingers and toes, a large body size (64.9 mm in SVL) and lacks mental glands. Broad dermal fringes are a putative morphological synapomorphy of the genus *Hyloscirtus* (Faivovich et al. 2005; Rivera-Correa and Faivovich 2013), and the other two characteristics suggest that this specimen might be related to species included in the *Hyloscirtuslarinopygion* species group (Faivovich et al. 2005; Rivera-Correa and Faivovich 2013; Duellman et al. 2016)—although some species of the group have mental glands, e.g., *H.caucanus* (Brunetti et al. 2015). The specimen from Río Negro-Sopladora National Park shows some phenetic characteristics like those present in species of the northern clade of the *H.larinopygion* group. Species of the northern clade are morphological distinct from species of the southern clade as follows (condition for species of the southern clade in parentheses): HW/HL < 1.1 (HW/HL ≥ 1.1); longer snouts, usually EN/ED > 0.75 (EN/ED < 0.65); dentigerous processes of vomer in contact or slightly separated and having numerous vomerine teeth (widely separated, with few vomerine teeth); forearms robust and slightly thicker than upper arm (forearms and arms hypertrophied, similar to species of the *Hyloscirtusarmatus* species group); enlarged, broad, elliptical prepollex, hidden under thenar tubercle (protruding, curved prepollical spine); colouration on dorsum different from colouration on flanks, hidden surfaces of thighs and venter (coloration similar on dorsal, flanks and venter) (Fig. 1). The distinction between both clades of the *H.larinopygion* species group has been consistently identified in several studies (Almendáriz et al. 2014; Rivera-Correa et al. 2016; Rojas-Runjaic et al. 2018; Ron et al. 2018).

The specimen from Río Negro-Sopladora National Park shows a unique colouration pattern with pale coloured background and dark marks on dorsal, lateral, and ventral surfaces, while most species currently under the *H.larinopygion* group have dark-coloured backgrounds with dark or pale marks (except for *H.sarampiona* and some specimens of *H.larinopygion* and *H.psarolaimus*). While it is known from a single individual, we propose that the population of *Hyloscirtus* from the Río Negro-Sopladora National Park corresponds to an undescribed taxon, and we described it below.

### ﻿Systematics

#### ﻿ Hyloscirtus tolkieni sp. nov.

Taxon classificationAnimaliaAnuraHylidae

312214B4-BB22-5E70-B94A-FCBEF7B4DFEE

https://zoobank.org/0DA4A78A-D514-43FA-B5DA-8F5074F9E353

**Figure 2.**
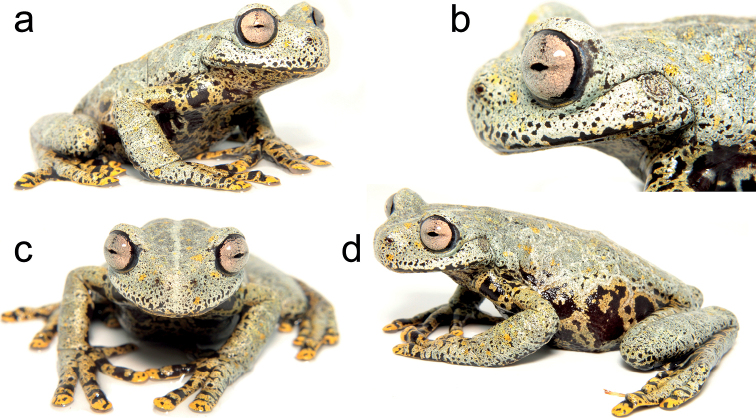
Holotype of *Hyloscirtustolkieni* sp. nov. (SVL = 64.9 mm) in life: **a** fronto-lateral view **b** lateral view of head **c** frontal view **d** dorso-lateral view. Photographs by Juan C. Sánchez-Nivicela.

**Figure 3.**
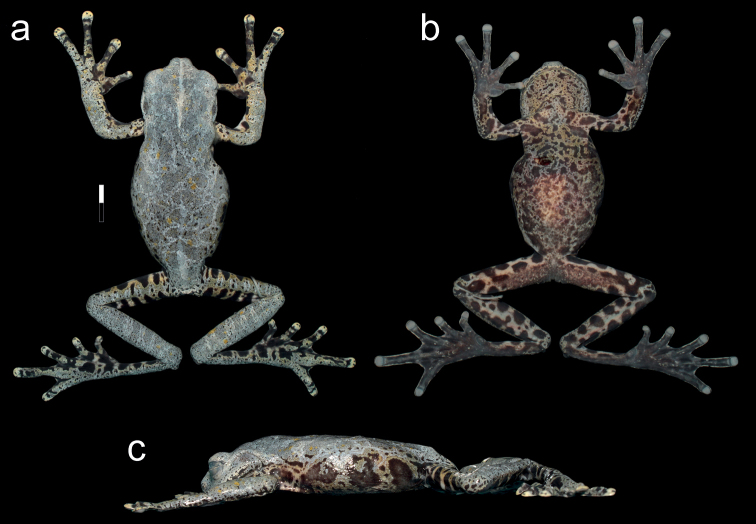
Holotype of *Hyloscirtustolkieni* sp. nov. in preservative **a** dorsal view **b** ventral view **c** lateral view. Scale bar: 1 cm.

**Figure 4.**
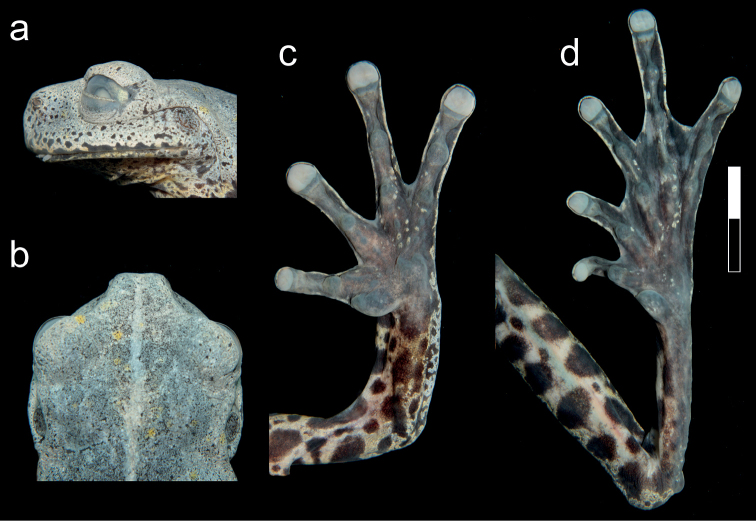
Details of *Hyloscirtustolkieni* sp. nov. in preservative **a** lateral view of head **b** dorsal view of head **c** ventral view of hand **d** ventral view of foot. Scale bar: 1 cm.

**Figure 5.**
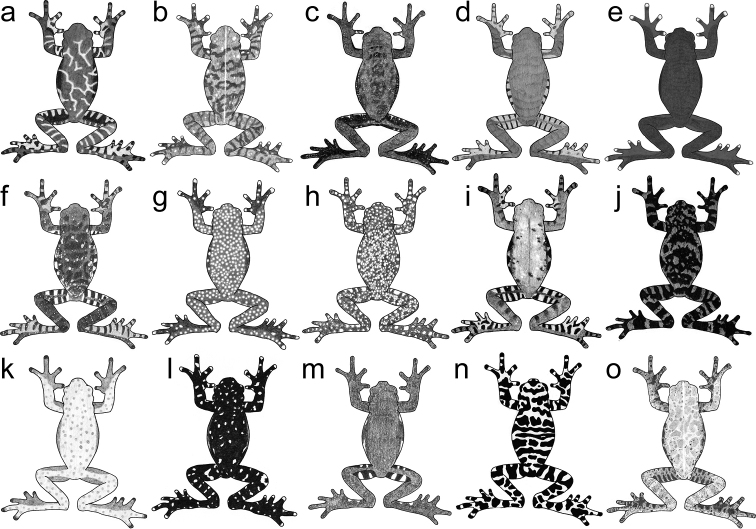
Dorsal colouration patterns in species of the northern clade of the *Hyloscirtuslarinopygion* species group **a***H.antioquia***b***H.caucanus***c***H.criptico***d***H.larinopygion***e***H.lindae***f***H.pacha***g***H.pantostictus***h***H.princecharlesi***i***H.psarolaimus***j***H.ptychodactylus***k***H.sarampiona***l***H.sethmacfarlanei***m***H.staufferorum***n***H.tigrinus***o***H.tolkieni* sp. nov. Illustrations by José M. Falcón-Reibán.

**Figure 6.**
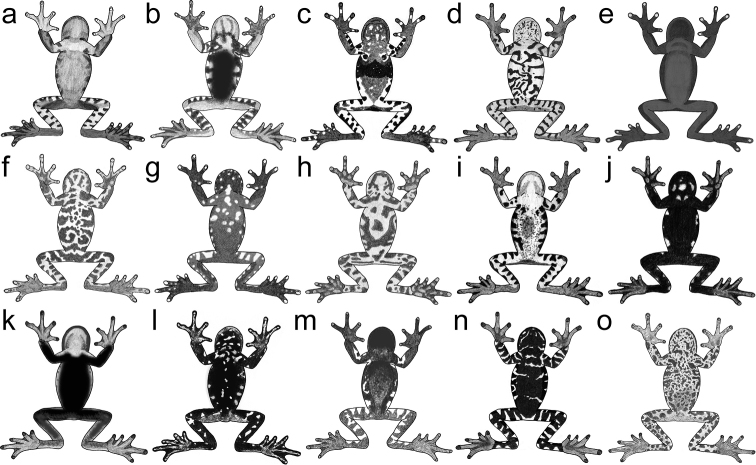
Ventral colouration patterns in species of the northern clade of the *Hyloscirtuslarinopygion* species group: **a***H.antioquia***b***H.caucanus***c***H.criptico***d***H.larinopygion***e***H.lindae***f***H.pacha***g***H.pantostictus***h***H.princecharlesi***i***H.psarolaimus***j***H.ptychodactylus***k***H.sarampiona***l***H.sethmacfarlanei***m***H.staufferorum***n***H.tigrinus***o***H.tolkieni* sp. nov. Illustrations by José M. Falcón-Reibán.

**Figure 7.**
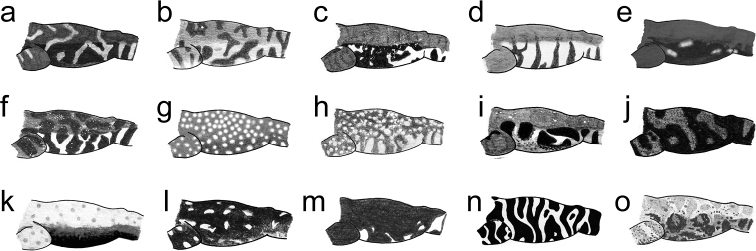
Flank colouration patterns in species of the northern clade of the *Hyloscirtuslarinopygion* species group: **a***H.antioquia***b***H.caucanus***c***H.criptico***d***H.larinopygion***e***H.lindae***f***H.pacha***g***H.pantostictus***h***H.princecharlesi***i***H.psarolaimus***j***H.ptychodactylus***k***H.sarampiona***l***H.sethmacfarlanei***m***H.staufferorum***n***H.tigrinus***o***H.tolkieni* sp. nov. Illustrations by José M. Falcón-Reibán.

Figs 2 , 3 , 4 , 5o , 6o , 7o English common name: Rio Negro Stream Treefrog Spanish common name: Rana de Torrente de Río Negro

##### Holotype

(Figs 2–4) ZSFQ-4142 (field number JCS-1613), adult female collected at Puente de Piedra (2°47'13"S, 78°36'16"W; 3190 m), Parque Nacional Río Negro-Sopladora, provincia de Morona Santiago, República del Ecuador, by José M. Falcón-Reibán, Juan C. Sánchez-Nivicela, and Tarquino Valverde, on 5 February 2020.

##### Diagnosis.

*Hyloscirtustolkieni* differs from other congeneric species by the following combination of characters: large body size (64.9 mm SVL in a single adult female); broad dermal fringes in fingers and toes; discs slightly expanded; head 7% wider than long; snout truncate in dorsal and lateral view; tympanic membrane and annulus evident, partially covered by supratympanic fold; dentigerous process of vomers slightly separated, with 9–13 vomerine teeth; forearm robust and slightly thicker than arm; discs slightly expanded; broad dermal fringes in fingers and toes; prepollex enlarged, hidden under thenar tubercle and not projected into a prepollical spine; subarticular tubercles on hands and feet rounded and poorly projected; calcar tubercle present; dorsum greyish-green, with paler-hued reticulum, yellow spots and black speckles; throat, venter, flanks and hidden surfaces of limbs yellow with large black blotches and spots; fingers, toes and webbing yellow with black bars and spots; iris pale pink with black periphery, sclera greyish-blue, and nictitating membrane yellow (Figs 2–4).

*Hyloscirtustolkieni* is readily distinguishable from all other species of *Hyloscirtus* by its greyish-green dorsum with paler reticulations, yellow spots, and black speckles. Based on its colouration pattern, *Hyloscirtustolkieni* (characteristics in parentheses) is easily differentiated from all other species of the northern clade of the *Hyloscirtuslarinopygion* species group (Figs 5–7) as follows: Dorsal surfaces of *H.antioquia*, *H.caucanus*, *H.criptico*, *H.larinopygion*, *H.lindae*, *H.pacha*, *H.pantostictus*, *H.princecharlesi*, *H.psarolaimus*, *H.ptychodactylus*, *H.sethmacfarlanei* and *H.staufferorum* are dark or light brown or black with or without paler or darker marks, *H.sarampiona* is pale olive green with orange spots, and *H.tigrinus* is green or yellow with thick black reticulum or stripes (greyish-green dorsum with paler reticulum, yellow spots and black speckles). Flanks of *H.antioquia*, *H.caucanus*, *H.criptico*, *H.lindae*, *H.pacha*, *H.pantostictus*, *H.princecharlesi*, *H.ptychodactylus*, *H.sarampiona*, *H.sethmacfarlanei* and *H.staufferorum* are dark brown or black with or without paler or darker marks; bluish grey or cream with dark bars, blotches, or spots in *H.larinopygion* and *H.psarolaimus*; and yellow or green with thick black stripes or reticulum in *H.tigrinus* (yellow flanks with large black blotches and spots). Fingers, toes and discs are dark brown in *H.criptico* and *H.staufferorum*; dark brown with orange discs in *H.lindae*; bluish grey with dark bars in *H.larinopygion*; dark brown with pale bars in *H.pacha*; black with pale discs in *H.caucanus* and *H.pantostictus*; black with orange or red spots in *H.princecharlesi* and *H.sethmacfarlanei*; cream with dark marks in *H.psarolaimus*; black with reddish-brown marks in *H.ptychodactylus*; dark olive green with orange dots in *H.sarampiona*; and yellow or green with black marks in *H.tigrinus* (yellow with black marks). Irises of *H.criptico*, *H.pacha*, *H.pantostictus*, *H.princecharlesi*, and *H.staufferorum* are dark grey or brown without reticulations; grey with dark grey reticulations in *H.sethmacfarlanei*; grey with burgundy reticulations in *H.antioquia*; pale yellow with brown reticulations in *H.caucanus*; golden with black reticulations in *H.larinopygion*; dark brown with minute grey flecks in *H.lindae*; dull bronze with black reticulations in *H.psarolaimus*; pale blue in *H.ptychodactylus*; gold with black reticulations in *H.sarampiona*; and light grey or yellow with black reticulations in *H.tigrinus* (pale pink with very thin, almost imperceptible, reticulations). Snout rounded in dorsal view in *H.antioquia*, *H.caucanus*, *H.larinopygion*, *H.lindae*, *H.pacha*, *H.pantostictus*, *H.psarolaimus*, *H.sarampiona*, *H.staufferorum*, and *H.tigrinus* (truncated). Vomerine teeth 12–20 in *H.antioquia* and 21–25 in *H.staufferorum* (9–13). Calcar tubercles absent in *H.princecharlesi* (present).

*Hyloscirtustolkieni* has non-protruding prepollex and narrower head (HW/HL = 1.07), more vomerine teeth (9–13), and thinner forearms than species of the southern clade of the *H.larinopygion* species group (including *H.condor*, *H.diabolus*, *H.hillisi* and *H.tapichalaca*), which have protruding prepollical spines, wider heads (HW/HL ≥ 1.10), less vomerine teeth (2–6), and hypertrophied forearms. Also, all species of the southern clade of the *H.larinopygion* species group are dark-coloured dorsally and ventrally.

*Hyloscirtustolkieni* differs from species of the *H.armatus* species group by the absence of clusters of keratinised spines on the prepollex and the proximal ventral surface of the humerus (present in *H.armatus* and *H.charazani*), non-expanded prepollex (expanded in *H.armatus* and *H.charazani*), robust but not hypertrophied forearms (hypertrophied in *H.armatus* and *H.charazani*), and absence of a skin fold in the proximoventral portion of upper arm (present in *H.armatus*, *H.charazani*, and *H.chlorostea*).

*Hyloscirtustolkieni* differs from species of the *H.bogotensis* species group, including *H.albopunctulatus* and *H.phyllognathus* that inhabit the eastern Andes of Ecuador, and from *H.jahni*, single member of its homonym group, by its larger body size with 64.9 mm in SVL (smaller in the *H.bogotensis* and *H.jahni* species groups with SVL<36 mm), greyish-green dorsum with paler reticulum, yellow spots and black speckles (green or brown dorsum with or without pale or dark spots and speckles and pale lines in the *H.bogotensis* and *H.jahni* species groups), ventral surfaces yellow with large black blotches and spots (venter cream or yellowish without dark marks in the *H.bogotensis* and *H.jahni* species groups).

##### Description of the holotype.

Adult female (Figs 2–4), 64.9 mm SVL, body robust. Head wider than long (HW/HL = 1.07, HW/SVL = 0.31, HL/SVL = 0.29); snout truncate in dorsal and lateral view; canthus rostralis rounded, distinct; loreal region slightly concave, nearly vertical; lips rounded, slightly flared; nostrils slightly protuberant, openings directed anterolaterally, located at level of anterior margin of lower jaw, area between nostril slightly concave; dorsal surface of internarial region concave; interorbital distance shorter than eye (IOD/ED = 0.91); eye prominent (ED/HL = 0.37, ED/EN = 1.33); tympanic membrane and annulus evident (TD/ED = 0.41); supratympatic fold prominent, extending from below eye across upper and posterior margins of tympanum towards posterior end of mouth and down to arm insertion; region between head and suprascapula slightly depressed; dentigerous processes of vomer prominent, oval, in transverse position, between choanae, narrowly separated, left process with 9 vomerine teeth and right one with 13; choanae small, rounded, separated about 4× their maximum diameter; tongue cordiform, broad, attached to 80% of mouth floor; mental gland absent (Figs 2–4).

Skin on dorsum shagreen, throat slightly granular, flanks and venter granular, posterior surfaces of limbs strongly granular; pectoral fold absent; cloacal opening directed posteroventrally at upper level of thighs; supracloacal flap present; two pairs of swollen, thick, vertical, pericloacal folds.

Forearms robust, slightly thicker than arms, not hypertrophied; axillary membrane absent; ulnar fold present, covering dorsal surface of forearms; fingers long, with thick lateral fringes; discs round, slightly expanded; all discs with rounded pads, circumferential groove of each disc clearly defined; disc on Finger III wider than tympanum (Fin3DW/TD = 1.11); relative lengths on fingers I<II<IV<V; webbing formula: **III**3-–3-**IV**; palmar surface with deep grooves; subarticular tubercles round and poorly projected, distal tubercles larger; supernumerary tubercles small, rounded; thenar tubercle large, elliptical; palmar tubercle flat, bifid, same length as thenar; broad elliptical prepollex hidden under thenar tubercle (Figs 2–4).

Hindlimbs robust (TL/SVL = 0.48, FL/SVL = 0.48); small calcar tubercle present; short and thin inner tarsal fold; without outer tarsal fold or tubercles; inner metatarsal tubercle large, ovoid; outer metatarsal tubercle indistinct; toes long, with thick lateral fringes, bearing discs slightly smaller than those on fingers; relative lengths of toes: I<II<III=V<IV; Toe I with last phalange twisted inside on both feet; webbing formula: **I**2–2**II**1⅔–2½**III**2–3-**IV**3–2-**V**. Subarticular tubercles large, round; supernumerary tubercles low, round, and sparse (Figs 2–4).

##### Colouration in life.

Dorsal surfaces of head, body and limbs greyish-green, with thick paler-hued reticulum, yellow spots, and black speckles; head with a light greyish-green medial line; throat, venter and flanks yellow (more intense on the throat and turning greyish towards posterior end of venter) with large black blotches and spots; hidden surfaces of limbs yellow with transversely distributed black oval dots; fingers, toes and webbing yellow with black bars and spots; iris pale pink with black periphery, sclera greyish-blue, and nictitating membrane yellow (Fig. 2)

##### Colouration in preservative.

Same colouration patterns as described for the colouration in life, but greyish-green dorsal areas turned darker grey, yellow on venter and flanks turned golden-grey to grey (Figs 3–4).

##### Measurements of the holotype

**(in mm).**SVL=64.9, HL=18.8, HW=20.2, IND=4.6, IOD=6.3, EW=4.9, EN=5.2, ED=6.9; TD=2.8, TL=31.2, FL=30.9, Fin3DW=3.1.

##### Etymology.

The specific epithet *tolkieni* is in honour of the writer, poet, philologist, and academic John Ronald Reuel Tolkien (J.R.R. Tolkien, 1892–1973), creator of Middle-earth and author of fantasy works like “The Hobbit” and “The Lord of the Rings”. The amazing colours of the new species evoke the magnificent creatures that seem to only exist in fantasy worlds.

##### Distribution, natural history, and conservation status.

**Figure 8.**
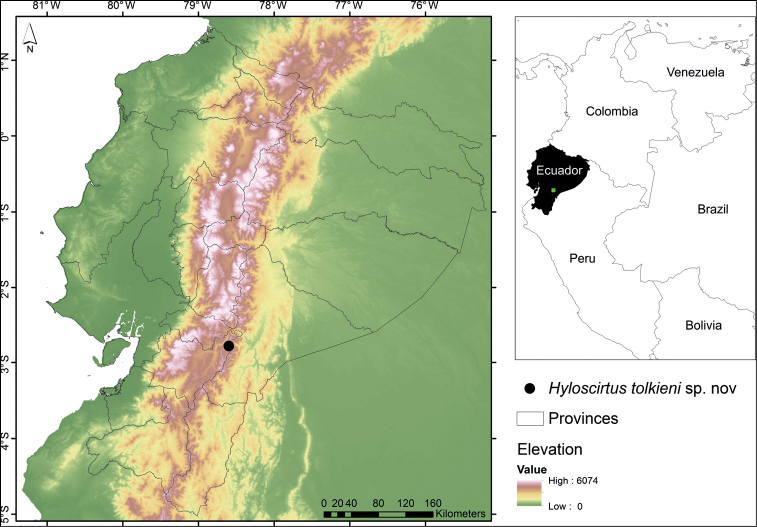
Map showing the type locality of *Hyloscirtustolkieni* sp. nov. at the Río Negro-Sopladora National Park, province of Morona Santiago, Republic of Ecuador.

**Figure 9.**
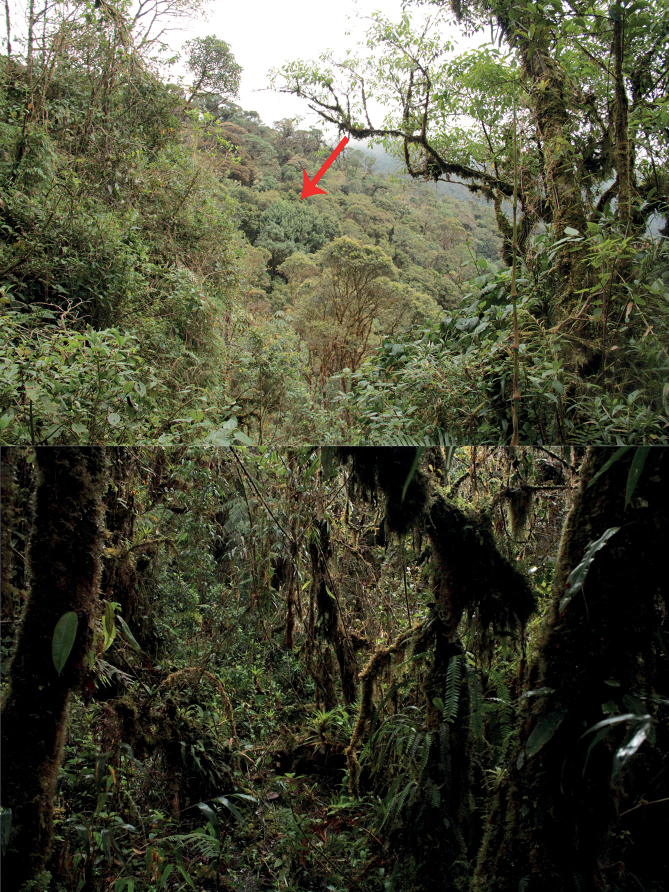
Habitat of *Hyloscirtustolkieni* sp. nov. General landscape (above, red arrow pointing to collection site); and at the collection site inside the forest (below). Photographs by Juan C. Sánchez-Nivicela.

*Hyloscirtustolkieni* is only known from its type locality on the southeastern slopes of the Cordillera Oriental of the Andes of Ecuador, at 3190 m elevation, in the Río Negro-Sopladora National Park, province of Morona Santiago (Fig. 8). The ecosystem in the area is High Montane Forest of the Eastern Cordillera of the Southern Andes of Ecuador (MAE et al. 2013). The holotype was active at night at 20:30 amidst tree branches, *c.* 5 m above ground and 8 m from the nearest stream (Fig. 9). It was found in sympatry with an undescribed species of *Pristimantis*.

Very few herpetological surveys have been conducted in the region, with James A. Peters being one of the few herpetologists that visited the area (Peters 1973). Our surveys were carried out over 13 effective days, and we could not detect additional individuals of *H.tolkieni*, despite focalised searches. The type locality of *H.tolkieni* is officially protected as part of the Río Negro-Sopladora National Park, a protected area created in 2018 where little habitat loss has occurred. Large, forested areas remain unstudied in the national park, and the species may have a wider distribution beyond the immediate surrounding of its type locality. In the absence of sufficient information to evaluate the conservation status and extinction risk of *H.tolkieni*, we propose that it be classified under the Data Deficiency category until more data is obtained (IUCN 2012, 2017; Ortega-Andrade et al. 2021). Urgent research and monitoring actions should be established to study its life history and ecology, population size and trends, survey new sites where additional populations may exist and evaluate if threats are impacting its long-term conservation, such as invasive species, emerging diseases, or climate changes.

### ﻿Key to the species of the northern clade of the *Hyloscirtuslarinopygion* species group

This key helps to identify adult female and male stream treefrogs of the northern clade of the *H.larinopygion* species group, using characters that can easily be observed in the field and lab (no dissections required). This key is probably not useful to identify juveniles and ontogenetic variation in many species of the group remains unknown. This key was expanded and corrected from the keys presented by Duellman and Hillis (1990) and Duellman and Coloma (1993). Colours in preservative are shown in parentheses.

**Table:** 

1a	Background dorsal colouration in shades of green or yellow (turning paler green or greyish cream) (Fig. 5k, n, o)	**2**
1b	Background dorsal colouration in shades of brown (Fig. 5a–j, l–m)	**4**
2a	Dorsum green or yellow (greyish cream) with thick black reticulum or stripes (Fig. 5n)	***H.tigrinus***
2b	Dorsum green (grey) without thick dark reticulum or stripes	**3**
3a	Dorsum pale olive green with orange dots (grey with cream spots) (Fig. 5k); venter black (Fig. 6k)	***H.sarampiona***
3b	Dorsum greyish-green with paler reticulum, yellow spots, and black speckles (green turns to grey) (Fig. 5o); venter, flanks, and hidden surfaces of limbs yellow (golden-grey) with large black blotches and spots (Fig. 6o)	***H.tolkieni***
4a	Venter (excluding throat) uniformly or predominantly black or dark brown (Fig. 5b, e, j, m)	**5**
4b	Venter mostly pale or dark with distinctive darker or paler markings	**8**
5a	Discs on fingers orange or yellow (pale)	**6**
5b	Discs on fingers dark	**7**
6a	Dorsum and venter dark brown (Figs 5e, 6e), discs on fingers orange in life	***H.lindae***
6b	Dorsum brown with dark brown transversal bars (Fig. 5b), venter black with pale marks (Fig. 6b), discs on fingers cream in life	***H.caucanus***
7a	Throat uniformly dark (Fig. 6m); hidden surfaces of limbs black with broad cream bars (Fig. 7m); iris dark brown	***H.staufferorum***
7b	Throat with irregular, large, pale spots (Fig. 6j); hidden surfaces of limbs black, sometimes with reddish-brown (Fig. 7j); iris pale blue in life	***H.ptychodactylus***
8a	Dorsum with orange or red (pale) circular dots on a dark background	**9**
8b	Dorsum without orange or red (pale) circular dots. If orange (pale) markings are present, they are in the form of flecks or blotches but not circular dots	**10**
9a	Discs on fingers yellow (white); venter black with white mottling on belly and orange dots (white) on the throat (Fig. 6g)	***H.pantostictus***
9b	Discs on fingers grey, venter black with pale yellow (cream) marbling or reticulation (Fig. 6h)	***H.princecharlesi***
9c	Disc on fingers with red spots (yellowish white); venter black with red (yellowish white) dots (Fig. 6l)	***H.sethmacfarlanei***
10a	Venter cream to brownish or dirty grey	**11**
10b	Venter dark brown with pale markings	**12**
11a	Venter dirty grey (Fig. 6a); dorsum brown with orange (grey) reticulation (Fig. 5a); flanks grey or black with yellow (cream) markings delimited with blue or pale-grey outline (Fig. 7a); iris grey with burgundy reticulations in life	***H.antioquia***
11b	Venter cream to brownish grey with diffuse dark spots and pale flecks (Fig. 6i); dorsum brown with small dark brown and cream flecks (Fig. 5i); flanks cream with vertical dark bars (Fig. 7i); iris dull bronze with black reticulations in life	***H.psarolaimus***
12a	Flank black with orange (cream) speckles and some white and brown blotches (Fig. 7c); venter mottled dark brown with orange (cream) speckles and pale marks (Fig. 6c)	***H.criptico***
12b	Flanks dark with pale reticulum or pale with black vertical bars	**13**
13a	Dorsum brown with small orange (cream) flecks (Fig. 5f); venter dark brown with bold cream reticulum (Fig. 6f); hidden surfaces of limbs black with narrow, vertical, cream bars (Fig. 7f)	***H.pacha***
13b	Dorsum brown with or without dark-brown reticulation (Fig. 5d); flanks, venter, and hidden surfaces of limbs white or light bluish grey (cream) with black bars or reticulation (Figs 6d, 7d)	***H.larinopygion***

## Supplementary Material

XML Treatment for Hyloscirtus tolkieni
